# Comparison of Corrosion Behavior of T91, 9Cr and 9CrAl ODS Steels in Liquid Pb

**DOI:** 10.3390/ma16062295

**Published:** 2023-03-13

**Authors:** Lingzhi Chen, Shuai Xu, Carsten Schroer, Haodong Jia, Zhangshun Ruan, Bo Qin, Zhangjian Zhou, Bin Long

**Affiliations:** 1 Institute of Reactor Engineering Technology, China Institute of Atomic Energy, Beijing 102413, China; 2 School of Materials and Chemistry, Southwest University of Science and Technology, Mianyang 621010, China; 3 Institute for Applied Materials-Applied Materials Physics, Karlsruhe Institute of Technology (KIT), 76344 Karlsruhe, Germany; 4 School of Material Science and Engineering, University of Science and Technology Beijing, Beijing 100083, China

**Keywords:** ODS steel, 9CrAl, liquid Pb, corrosion layer, dissolution

## Abstract

It is important to improve the liquid lead corrosion resistance of fuel cladding alloy for promoting the development of lead-based reactors. The corrosion behaviors of traditional T91 steel and similar oxide dispersion strengthen ODS-type steels with or without the addition of Al, and were examined and compared at 600 °C in static oxygen-controlled liquid Pb in this research. High-temperature liquid lead corrosion tests were carried out for 120 h, 240 h, 500 h, 1000 h, and 2000 h, respectively, for three prepared samples. After the experiment, the corrosion behavior was evaluated and compared mainly based on the aspects of appearance, corrosion depth, microstructure, and composition difference. It was found that just the ODS design did not show a positive effect on corrosion resistance, while the addition of Al is beneficial to improving the corrosion resistance of ODS steel. The maximum corrosion depth of 9CrAl ODS is only 51.8 μm after corrosion for 2000 h, which is much lower than that of 9Cr-ODS steel. A thin film containing Al/Cr formed in the corrosion area after adding Al in 9Cr ODS steel, which played a positive role in corrosion resistance.

## 1. Introduction

The lead-cooled fast reactor (LFR) has become one of the most promising advanced reactors because of its good neutron characteristics, chemical stability, thermal hydraulic characteristics, and the ability to transmutation of nuclear waste. The coolant of this kind of reactor, lead or lead–bismuth eutectic (LBE) alloy, may also have important application prospects in accelerator-driven systems (ADS) and fusion reactors.

The core components, especially the fuel cladding of LFR, work in a high-temperature, high-irradiation-damage, and severe corrosive environment, which requires excellent high-temperature creep resistance, strong irradiation resistance, and especially the ability to resist liquid metal corrosion [1]. Compared with other corrosive media, such as high-temperature water or gas, the liquid metal in lead-based reactors has serious dissolution corrosion to structural materials at high temperature, especially the elements of Ni and Cr [2]. The T91 ferritic/martensitic steel does not contain Ni and has a lower content of Cr compared to austenitic steels and, therefore, is considered an important candidate for lead-cooled reactors. However, the working temperature of traditional ferritic/martensitic steel is usually below 550–600 °C, which cannot meet the service requirements of the future lead-cooled reactor. If introducing very fine oxide particles with a high number density into the matrix of steel, the operation temperature can be increased to 700 °C [3]. The concept of ODS also can improve creep property and irradiation resistance obviously [4,5,6,7]; therefore, ODS steel is considered as a promising candidate structural material for advanced reactors [8].

Traditional steels will be severely corroded in a lead and/or LBE environment, especially when the temperature is higher than 500 °C. The high dissolution rate of the main elements of steel in liquid lead brings great hidden dangers to the safe service of the structure materials. One of the main approaches to fight lead corrosion is to form a protective layer on the surface of the materials by a reasonable control of the oxygen content in liquid lead [9]. For traditional steel, such as T91, Fe- and Cr-rich oxide layers will form during oxidation, but their structure is not dense enough to protect the matrix, especially at a high temperature. Forming a more stable and protective oxide layer on the surface of steel by optimizing the alloy design received more attention on the materials R&D of LFR in recent years. Al has a strong oxygen affinity; the standard Gibbs formation energy and required oxygen concentration of Al oxidation in lead is lower than those of the Fe, Cr, and Si elements, while the thermodynamic stability of the oxidation product Al_2_O_3_ is stronger. The formation of a continuous and dense Al_2_O_3_ film can reduce the further diffusion of oxygen into the matrix and migration of alloy elements to the coolant [10,11]. It is an important scientific method to form Al_2_O_3_ oxide film in situ by adding a strong oxide forming element Al. The alumina formation rate is controlled by the diffusion rate of oxygen ions through the oxide layer into the matrix, and the migration rate of metal ions through the oxide–liquid interface [10].

Some results on 14Cr ODS and 9Cr ODS steels showed that the fine-grain structure and high Cr content could increase the oxidation and corrosion resistance of ODS steels in liquid Pb with oxygen [12]. It has been reported that Al-containing ODS steel with a 16% Cr content has good resistance to LBE corrosion [13]. When Cr reaches a critical content, it can promote the formation of Al_2_O_3_ oxide film, which is called the "third element effect" [14,15,16]. Research also showed that the 12CrAl ODS steel also has good Pb-Li corrosion resistance [17]. These studies indicate that the addition of Al may also improve the corrosion resistance of ODS steel even if the Cr content is not very high. The special microstructure characteristics of ODS steel, such as the ultra-fine grain structure, a large number of grain boundaries, and dispersed nano-oxide particles, are different from those of traditional steel, which have a significant impact on its corrosion resistance [18]. However, whether the Al-containing ODS steel still shows better lead and LBE corrosion resistance when the Cr content is low has not been clearly reported. There are few reports on the compatibility of ODS steels with liquid lead, and even fewer reports on Al-containing ODS steels. The detailed effect of the specific micro structural state of ODS steel on the oxidation or dissolution process is still not well-known.

This work focuses on the investigation of the compatibility between liquid lead and candidate cladding materials with a low Cr content for LFR, especially focusing on the effect of an Al addition on the liquid lead resistance of ODS steel with a low Cr content. The corrosion behaviors of T91 steel, 9Cr ODS steel, and a new ODS steel with an Al addition in liquid lead were studied and compared. The effects of the steel manufacturing process and composition on the corrosion behavior and kinetic process of steels were discussed. 

## 2. Materials and Methods

### 2.1. Materials and Specimens

T91 is commercial material in forged state. ODS steels used in this study were prepared by mechanical alloying combined with hot isostatic pressing (HIP). Argon-atomized iron-chromium alloy powder with a size of −200 mesh, high-purity Ti powder, high-purity Al powder, and nano-sized Y_2_O_3_ powder (30 nm, 99.99%) were mixed and mechanical alloyed (MA) in a high-purity argon atmosphere using an omnidirectional planetary ball mill, then the MA powder was sintered by HIP, and the HIPed samples were forged. This process has been explored and reported in our previous works [19,20].

The specimen used in the corrosion test is a cylinder with a blind hole. Different specimens can be connected through the blind hole with a ceramic rod. The size of the cylinder is Ø8 mm × 15 mm, which was made from block-forged steels by machining, and then finely polished to make the surface of specimen smooth. The specimen cutting is required so that the corrosion liquid surface is parallel to the grain elongation direction. The chemical compositions of specimens are shown in Table 1.

### 2.2. Test Methods

**Figure 1:**
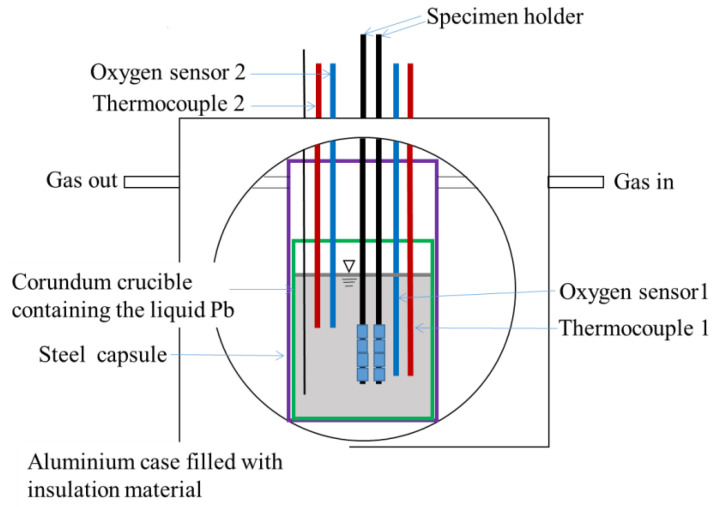
Schematic illustration of the device for Pb corrosion tests.

Tests were performed under different corrosion time for about 120 h, 240 h, 500 h, 1000 h, and 2000 h at 600 °C, respectively. The specimen’s codes are shown in Table 2. Figure 1 shows the device used for the exposure test in liquid Pb. The corrosion specimens were immersed in static lead. The Pb is contained in a corundum crucible. Two pairs of oxygen sensor and associated thermocouple monitor the dissolved oxygen concentration and temperature in two positions inside the crucible. The aluminum case contains a stainless-steel capsule that provides two 20 mm inner diameter ports for sample introduction. During the tests, it shall be ensured that all specimens are in the constant temperature zone of the test section, the local temperature difference in the test section shall not exceed +/− 1(2) °C, and the temperature difference between the average temperature and the nominal temperature of the test section shall not exceed +/− 3 °C.

Liquid Pb was contained in corundum crucible with a dimension of Øi102 × 145 mm. The inventory of liquid Pb in the crucible is 900 cm^3^, which corresponds to about 9 kg. The oxygen concentration in Pb was controlled using a flowing gas mixed with Ar, H_2_, and air. The oxygen contents in Pb during the tests were in the range of Co~10^−6^ wt% to Co~10^−7^ wt%. 

### 2.3. Post-Test Examinations

After the corrosion tests, the specimens were taken out for natural cooling. The specimens were cold-mounted with epoxy resin. The cross-sections of the specimens were observed and analyzed using optical and scanning electron microscopes after fine polishing. Corrosion performance of different materials in lead were evaluate by measurement of corrosion area, average depth, and maximum depth on the specimens [21].

Scanning electron microscopy SEM (Zeiss Gemini 300, Oberkochen, Germany) with EDS was used to observe and analyze the morphology and structure of the corrosion product. First, we made a mark on the specimen every 30°, marked 12 times for the whole sample, then measured along the marks under the SEM. The information of entire circular cross-section of specimen can be obtained by analyzing the numerical change of the sample after corrosion. Corrosion kinetics are assessed by comparing the corrosion data change relationship as a function of time. Electron micro-probe EPMA (EPMA-1600, Tokyo, Japan) was used to quantitatively analyze the composition of corrosion products.

FIB was used to prepare the TEM specimens after corrosion test, and TEM (JEM 2200FS, Osaka, Japan) was used to observe the morphology of corrosion product in detail. Specimen containing corrosion edge region area were prepared by focused ion beam FIB extraction technology, then the morphology was analyzed by transmission electron microscopy. The prepared specimen was analyzed by EDS super-spectroscopy using a spherical aberration transmission electron microscope equipped with a super-spectroscopy probe.

## 3. Results and Discussion

### 3.1. Appearance

**Figure 2:**
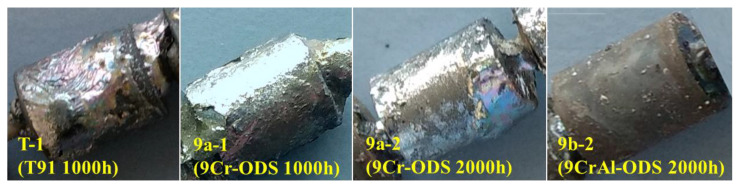
Appearance of the specimen after etching in liquid Pb.

After the corrosion test, the T91, 9Cr, and 9CrAl-ODS specimens were taken out for natural cooling. The appearance of the obtained specimens is shown in Figure 2. It can be seen clearly that the surface morphology and color of the three materials are quite different. The color of T91 is more abundant with an obvious metallic luster, which may be due to the formation of more complex oxidation products and adhered lead. Most of the surface of 9Cr ODS is gray-white with a strong metallic luster, which should be covered with metallic lead. The color of the 9CrAl ODS surface is gray and without metallic luster, which indicated that almost no metallic lead adhered on this material. Furthermore, the uniformity of surface color and roughness of the three specimens are different. The surfaces of T91 and 9Cr ODS steels are quite in-homogeneous, while 9CrAl ODS shows a relatively smooth surface. The different surface color and morphology imply the different corrosion behavior in liquid lead of different materials.

### 3.2. Quantification

A microscopic observation and quantitative analysis of the corroded specimens were performed by SEM-EDS. Both corrosion types of dissolution and oxidation can be found for all samples. The dissolution corrosion area, average depth, and maximum depth were measured and compared, as shown in Table 3. After corrosion for 1000 h, T91 shows a 41% proportion of the dissolved area; the maximum corrosion depth and average depth were 52.04 μm and 32.2 μm, respectively. After corrosion for 1000 h, the dissolved area of 9Cr ODS is 50%, even larger than that of T91; the maximum corrosion depth is 53.50 μm, similar to T91, and the average corrosion depth is 17.27 μm, much smaller than T91. After 2000 h of corrosion, the dissolved corrosion area of both 9Cr and 9CrAl-ODS steel are 58%. With the addition of Al in 9Cr ODS steel, the maximum corrosion depth decreased from 86.8 μm to 51.8 μm, and the average corrosion depth decreased from 48.3 μm to 40.9 μm. Although the addition of Al did not reduce the proportion of the corrosion area on the surface, the maximum corrosion depth decreased obviously, indicating that the addition of Al shows a great impact on the corrosion mode and corrosion products.

**Figure 3:**
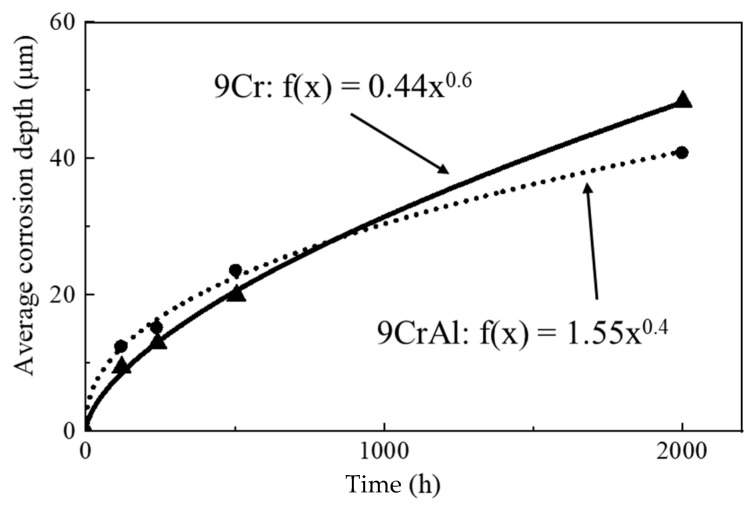
Corrosion kinetic process curves of 9Cr and 9CrAl ODS steels.

Based on quantitative corrosion data of the experimental specimens after exposure for 120 h, 240 h, 500 h, and 2000 h, the change of the average corrosion depth along with the exposure time can be simulated as shown in Figure 3. The obtained corrosion kinetic equations of 9Cr and 9CrAl ODS are shown in Equations (1) and (2), respectively:

(1) $$f\left(x\right)=0.44x^{0.6}$$

(2) $$f\left(x\right)=1.55x^{0.4}$$

It can be seen from the figure that, at the beginning, the average corrosion depth of 9Cr and 9CrAl are not much different. With the increase of corrosion time, the corrosion depth of 9CrAl will be smaller than that of 9Cr. The addition of Al has an obvious positive effect on the corrosion resistance of 9Cr ODS steel.

### 3.3. Micro-Structure Observation

#### 3.3.1. T91

**Figure 4:**
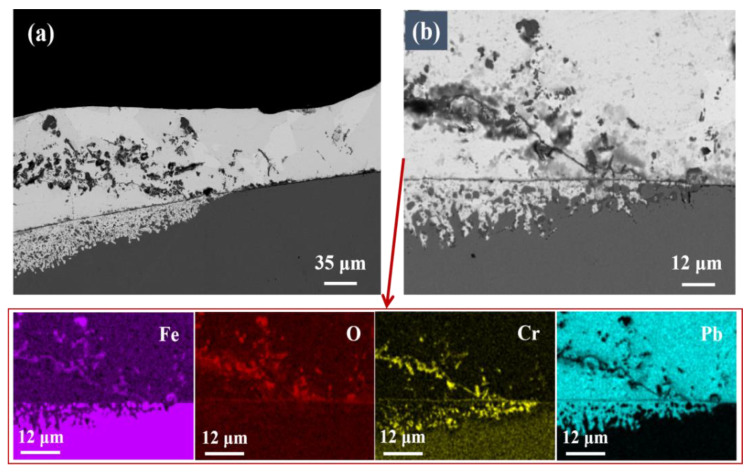
SEM images of T91 steel: (**a**) low magnification, (**b**) high magnification, and EDS element mapping.

**Figure 5:**
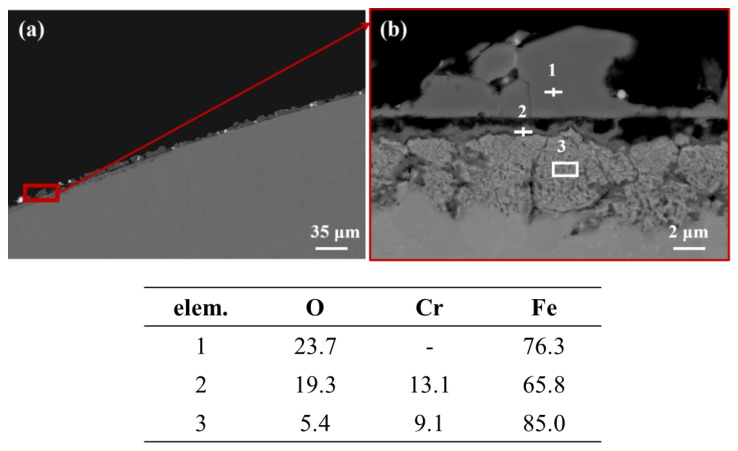
SEM images and EDS point scan of T91: (**a**) low magnification, (**b**) high magnification.

The cross-sectional morphology of the T-1 specimen was observed under SEM, as shown in Figure 4. Most areas showed dissolution corrosion. The white liquid metal Pb adhered on the surface and penetrated into the matrix, although Cr-rich oxides may form along the original boundary. In some areas, there was no obvious Pb infiltration, and there was discontinuous particulate matter that bulged outwards outside the original boundary, as shown in Figure 5. An oxide layer should be formed according to EDS analysis. Area 1 is Fe_3_O_4_ oxide growing out; area 2 is (Fe,Cr)_3_O_4_ oxide. Zone 3 is the inner oxidation zone (IOZ) formed by the diffusion of O into the matrix interior. During the test, the T91 sample mainly suffered from dissolution corrosion, which is consistent with the reported results based on LBE corrosion research [22]. Usually, the formation of Cr-rich oxides can play a positive role in anti-corrosion, but the formed oxide layer is a discontinuity for the investigated T91 material due to the low Cr content, and therefore fails to play a protective role against corrosion. 

#### 3.3.2. 9Cr ODS

**Figure 6:**
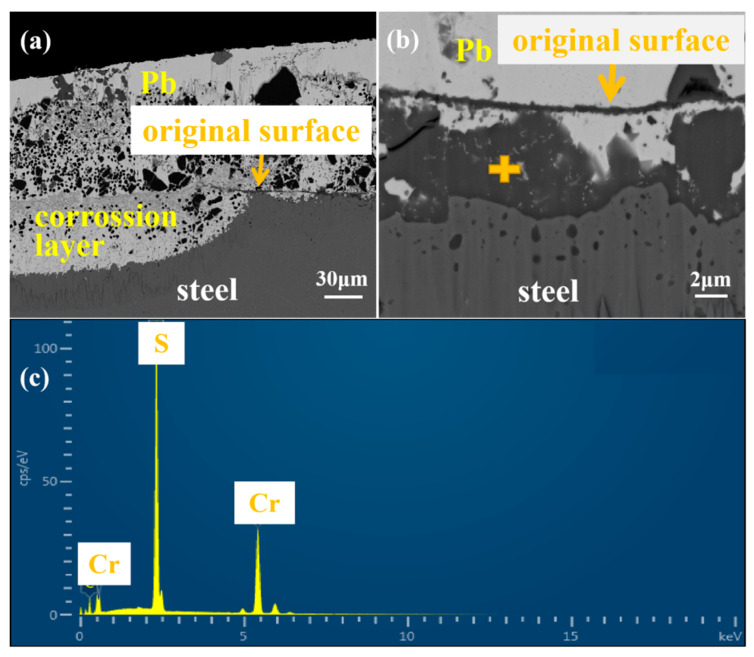
SEM images of 9Cr-ODS steel: (**a**) high magnification, (**b**) low magnification, and (**c**) EDS point scan.

According to SEM scanning of the cross-section of 9a-2, more than half of the area showed obvious dissolution corrosion, and a thick layer of solid Pb was adhered. As shown in Figure 6a, the left part shows the severe penetration of Pb, and the depth of the corrosion layer exceeds 60 μm. Corrosion on the right part is relatively slight, where a linear, flake-like, and gray-black product can be found close to the edge of the specimen. Combined with the shape of the incompletely dissolved part of the specimen and literature reports, it can be judged that this linear product is the original boundary of the sample [23].

The morphology and composition of the flaky gray-black product were analyzed at a higher magnification, as shown in Figure 6b,c; the EDS energy spectrum showed that it mainly contained Cr and S elements. The sulfide may be derived from the reaction of S with the sample components. This kind of Cr\S-sulfide product shows a certain protective effect to prevent the attack of liquid Pb. However, it is not continuous and its density is not high enough to completely resist the penetration of Pb. The white Pb continues to penetrate the material through the sulfide. Inside the Pb-attached area, there are many black particles with different sizes and regular edges, which were detected as C particles. As Pb is softer than the matrix, the C particles on sandpaper were embedded into the solid Pb during the polishing process. 

Comparing the cross-section morphology between T91 and 9 Cr ODS, as well as corrosion data listed in Table 3, it can be concluded that in the high-temperature liquid Pb at 600 °C, the fine-grained structure of 9Cr ODS steel will accelerate the dissolution of components compared with T91 with a similar composition, as there is not enough protective oxide film formed on the surface of 9Cr ODS steel.

**Figure 7:**
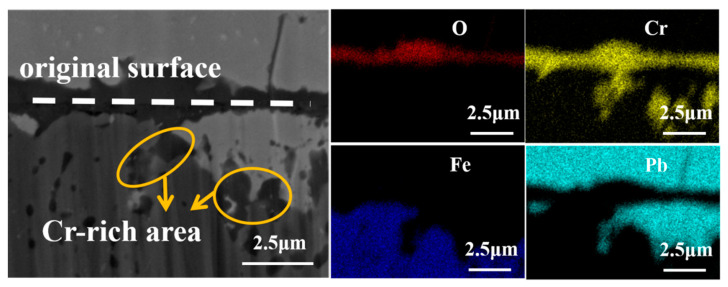
EDS mapping of linear corrosion products of 9Cr-ODS steel.

A further SEM observation at high magnification and EDS scan analysis were performed on the slight corroded area of 9Cr ODS, as shown in Figure 7. A thin local distributed layer with a thickness of 1 micron can be found along the original boundary, which separated the corrosion layer and Pb attached to the surface of the sample. EDS results show that this layer was rich in Cr and O elements, and the distribution of Cr and O is consistent, indicating that it was an oxide film rich in Cr_2_O_3_. However, this film is not continuous and dense enough due to the low Cr content of the material; therefore, it cannot play a protective role for the long-term corrosion resistance. As a result, there is white Pb under this layer, and there is an IOZ region at the boundary between the penetrated Pb and the matrix, which is a discontinuous internal oxidation product rich in Cr. This is consistent with the reported results of 9Cr ODS steel in flowing LBE. Under the condition of 10^−6^ wt% oxygen concentration, IOZ internal oxidation was always formed [24]. White Pb that appeared under the linear product showed that Pb continues to penetrate into the matrix through the linear oxide film. The content of Cr in 9Cr ODS is not high enough to form a continuous protective oxide film on the surface. On the contrary, the fine-grained structure accelerated the dissolution of components into liquid metal [12]. Under the experimental conditions, 9Cr ODS steel is mainly subject to dissolution corrosion. 

#### 3.3.3. 9CrAl ODS

**Figure 8:**
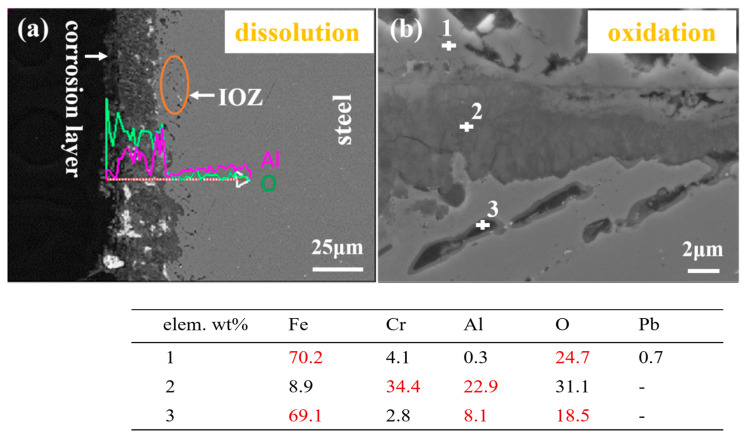
SEM images and EDS point scan of 9CrAl-ODS corroded for 2000 h: (**a**) low magnification, (**b**) high magnification.

According to the cross-section SEM information of 9b-2, the corrosion product morphology and structure were changed significantly compared with 9a-2 steel. As shown in Figure 8a, the corrosion depth is more than 30 μm, including the penetration of Pb and the IOZ region of the scattered oxide particles. The depth of the IOZ region is about 10 μm. SEM observation did not find a line-like corrosion product similar to 9Cr ODS. However, white Pb directly penetrated into the material to form a corrosion layer. EDS line scanning found that the content of the Al element in the corrosion layer was higher than that of the matrix, and the increasing trend of the Al and O peaks was consistent, which preliminary indicated that it was an oxide containing Al. From 9Cr to 9CrAl-ODS, with the addition of Al, the corrosion product changed from a Cr\S-containing structure to an obvious Al-containing structure.

An oxide layer was formed in some areas of 9CrAl, where no penetration of liquid metal was found, as shown in Figure 8b. The oxide scales can be divided into two parts: one is a thin gray layer that grows outward, and the other is a gray-black layer that grows inward. According to EDS analysis, the components’ mass percentage of region 1 (gray product growing out) is 70.2% Fe, 24.7% O, 4.1% Cr, 0.3% Al, and 0.7% Pb, which should be Fe_3_O_4_ with a magnetite structure. Region 2 (inward gray-black product) has a composition content of 8.9% Fe, 31.1% O, 34.4% Cr, and 22.9% Al, which is an inwardly growing complex mixture of (Fe,Cr)_3_O_4_ and (Fe, Al)_3_O_4_ with a spinel structure. The Fe content in this structure is relatively low due to the high solubility of Fe in Pb. For region 3, the composition content of Fe is 69.1%, O is 18.5%, Cr is 2.8%, and Al is 8.1%, which should be internally oxidized Al_2_O_3_. At 600 °C, O diffused rapidly inward in the matrix and reacted with Al to form Al_2_O_3_ particles, which are distributed discontinuously to form the IOZ region.

From 9Cr to 9CrAl-ODS, it is obvious that with the addition of Al, a thick oxide layer was formed in some areas, which can effectively reduce the corrosion. However, the combination of 4% Al and 9% Cr still failed to form a full continuous oxide scale. Since the binding ability of Al and O is stronger than that of Cr, the product in the IOZ region is transformed from Cr_2_O_3_ to Al_2_O_3_. The fine grain structure of ODS steel promoted element diffusion, and the IOZ of 9CrAl was more obvious.

**Figure 9:**
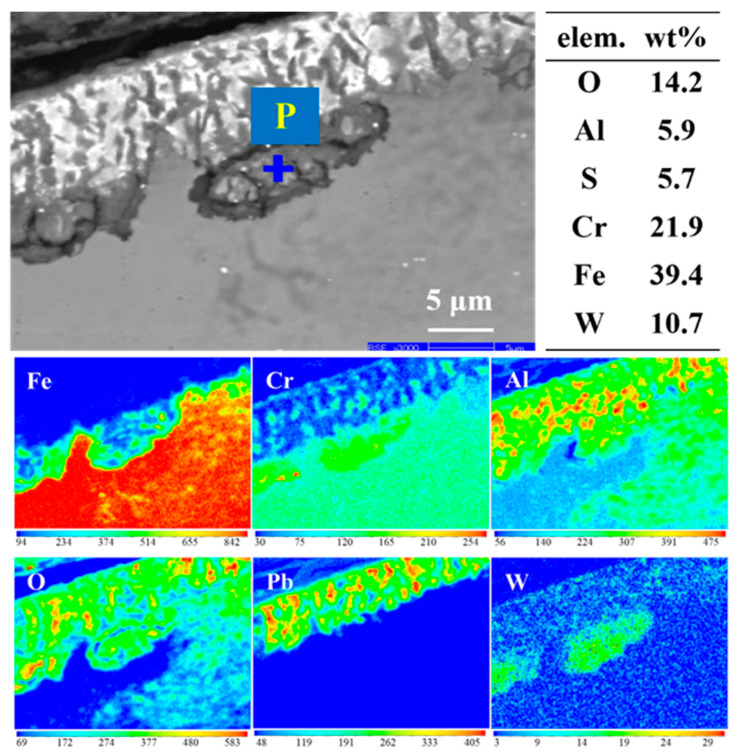
Electron probe analysis of 9CrAl-ODS steel corrosion area: topography, point scan, and element mapping.

Electron probe tests were performed on areas with severe dissolution corrosion, as shown in Figure 9. It can be seen from the EDS analysis of P point and surface scan that Pb penetrated into the interior of the material, which was consistent with the SEM analysis. The point analysis of the large black substances shows that the content of Cr, Al, and S are also high. It is speculated that the product structure may contain S, Cr, and Al, which is similar to the formation of sulfide in the 9Cr-ODS corrosion layer, indicating that the sample did not form a full protective oxide film at an oxygen concentration of 10^−6^ wt%. There are discontinuous Al-rich oxides at the interface between the penetrated Pb and matrix, which is the IOZ region formed by the internal oxidation of Al_2_O_3_. There is also a W-rich phase at the interface between the Pb penetration zone and the matrix, which may be the segregation of W in the long-term high-temperature experiments.

**Figure 10:**
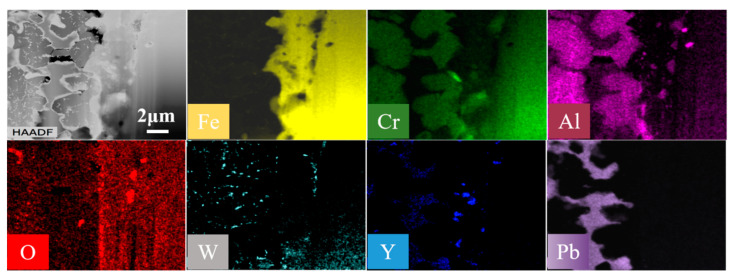
STEM/EDS super-spectroscopy mapping of the corroded region of 9CrAl-ODS steel.

The Pb penetration area was selected for FIB extraction. The FIB cutting position included both the corroded area and the substrate. The thickness was about 100–120 nm with a width of about 15 μm × 20 μm. The composition of the FIB specimen was characterized by STEM-EDS super spectroscopy, as shown in Figure 10. A large amount of Fe dissolved into liquid lead from the corrosion layer, resulting in Pb penetration and substitution. STEM also showed that the gray irregularities remaining in the corrosion zone were structures containing Al and Cr, with black nano-precipitates inside. The enriched regions of Al and Cr are consistent, which is consistent with the results of the electron probe. Possibly a compound containing Al and Cr was formed. Compared to the matrix, this substance is not easily dissolved by liquid lead. There were fewer corrosion products at the boundary between the substrate and the corrosion layer. Near the matrix boundary, some regions were also depleted of Fe. The presence of Al and O elements at the boundary between the matrix and the etched layer is highly coincident, which corresponds to the internal oxidation region of Al_2_O_3_.

At the boundary between the corrosion layer and the substrate, some fine particles rich in Al, Y, and O were found. It was speculated that they are Y-Al-O oxide particles dispersed in ODS steel [25], which have high thermal stability [8]. It also showed that the oxide dispersed particles were very stable during the corrosion process in this test. At the same time, obvious aggregation and segregation of W-rich particles were also found in the gray irregularity parts. This may be due to the dissolution of major elements in the steel and high stability of refractory metal in liquid Pb.

#### 3.3.4. Corrosion Resistance Mechanism of 9CrAl ODS

The fine micron grain size is the main difference of 9Cr ODS steel prepared by the powder metallurgy route and melted T91 steel. In this experiment, 9Cr ODS did not show superior corrosion resistance compared to T91, as the content of Cr is not high enough to form a protective oxide layer. When dissolution is the main corrosion mechanism, the large number of grain boundary channels in the fine-grained structure of 9Cr-ODS steel will accelerate the dissolution of alloy elements. At 600 °C, both the diffusion rate of Fe and Cr and their dissolution rate in Pb are high. Therefore, the rate of forming the protective oxide film becomes very important. As the sequence of oxygen affinity ability is Al > Cr > Fe, the Al_2_O_3_ oxidation film can be formed after adding Al. The oxidation resistance of Al_2_O_3_ is better than that of Cr_2_O_3_, which results in the corrosion resistance of 9CrAl ODS steel being significantly stronger than that of 9Cr ODS steel. 

**Figure 11:**
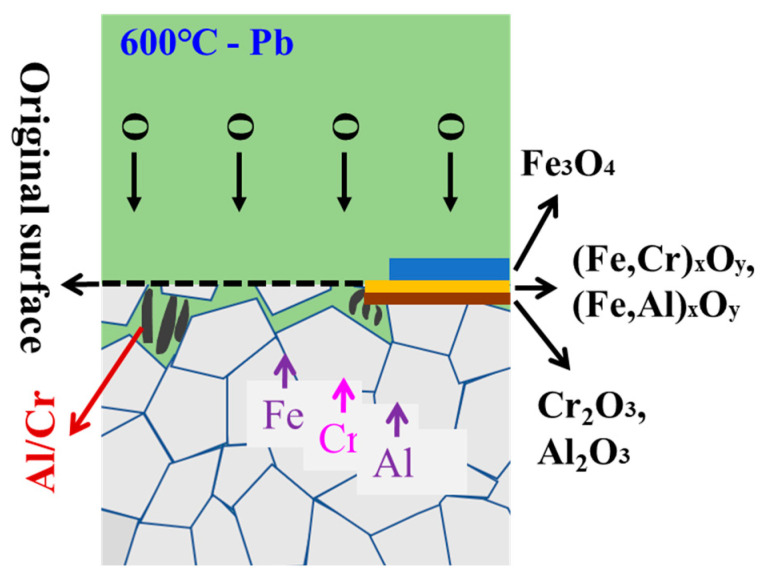
The corrosion resistance mechanism of 9CrAl ODS steel.

The corrosion products of 9CrAl ODS steel are more complex. The corrosion mechanism can be shown in Figure 11. At the initial stage of corrosion, oxidation products mixed with (Fe, Cr)_x_O_y_ and (Fe, Al)_x_O_y_ were formed at the interface between the alloy and liquid lead. Then, the continuous diffusion of Fe can form outward growing Fe_3_O_4_. The internal oxide layer is a denser and discontinuous oxidation product rich in Cr and Al. At the same time, a large amount of corrosion products with the Cr/Al structure were found in the Pb penetration area. For 9CrAl ODS steel, as the Cr content does not reach the critical concentration for the formation of a continuous oxide film, the "third element effect" is not obvious, Al undergoes internal oxidation to generate local Al_2_O_3_, the fine grain structure accelerates the diffusion of O to the material, and its internal oxidation IOZ region is more obvious than 9Cr ODS. However, the locally formed oxide film can still reduce the corrosion ability of liquid lead and greatly reduce the corrosion depth.

## 4. Conclusions

The corrosion behavior of T91, 9Cr ODS, and 9CrAl ODS in liquid Pb was tested and compared after exposure up to 2000 h at 600 °C. The main conclusions obtained are as follows:

1. The surface color and luster of the different corrode materials were quite different. The color of T91 is abundant with a metallic luster due to the formation of complex oxidation products and attached lead. Most of the surface of 9Cr ODS is gray-white with a metallic luster due to the covered metallic lead. The color of the 9CrAl surface is quite uniform without metallic luster;
2. The corrosion of 9Cr ODS is slightly heavier than that of T91 after corrosion for 1000 h. An oxide layer that grows outward is locally formed outside of T91, which plays a certain positive role in corrosion resistance, while the fine-grained structure of ODS steel promotes the accelerated dissolution of Fe and Cr, resulting in a lower rate of formation of oxidation products;
3. The maximum corrosion depth of 9CrAl ODS is 51.8 μm after corrosion for 2000 h, which is much lower than that of 9Cr-ODS steel. The reduction ratio is about 40%. Although the addition of Al cannot prevent dissolution corrosion for 9Cr ODS steel completely, it shows a positive impact on the corrosion mode and corrosion products due to the formation of a protective product containing Al/Cr.

## Figures and Tables

**Table 1:** Chemical compositions of specimens, wt.%.

Materials	Fe	Cr	Si	Ti	W	Y	O	N	C	Al	Mo	Mn
9Cr-ODS	Bal.	8.9	0.24	0.45	1.80	0.15	0.16	0.02	0.06	-	-	-
9CrAl-ODS	Bal.	8.9	0.24	-	1.80	0.15	0.18	0.02	0.06	4.50	-	-
T91	Bal.	8.5	0.28	0.001	0.001	-	-	-	0.08	0.015	0.84	0.59

**Table 2:** Coding list of specimens.

Materials	T91	9Cr-ODS	9CrAl-ODS
1000 h	T-1	9a-1	-
2000 h	-	9a-2	9b-2

**Table 3:** Corrosion data list.

	T-1	9a-1	9a-2	9b-2
Corrosion area	41%	50%	58%	58%
Average depth (μm)	32.2	17.27	48.3	40.9
Maximum depth (μm)	52.04	53.5	86.8	51.8

## Data Availability

Additional data can be obtained upon reasonable request from the corresponding author.
